# Comparative analysis of the effects of blade plate retention versus removal on paediatric bone remodelling following proximal femoral osteotomy

**DOI:** 10.1007/s00402-025-06058-6

**Published:** 2025-11-27

**Authors:** Emmanuel Eghan-Acquah, Alireza Y. Bavil, Henry P. J. Walsh, Martina Barzan, Stefanie Feih, Christopher P. Carty

**Affiliations:** 1 The Australian Centre for Precision Health and Technology (PRECISE), Griffith University, Gold Coast, Australia; 2 Advanced Design and Prototyping Technologies (ADaPT) Institute, Griffith University, Gold Coast, Australia; 3 School of Medicine and Dentistry, Griffith University, Gold Coast, Australia; 4 School of Engineering and Built Environment, Griffith University, Gold Coast, Australia; 5 Department of Orthopaedic Surgery, Children’s Health Queensland Hospital and Health Service, Gold Coast, Australia

**Keywords:** Paediatrics, Orthopaedics, Computational modelling, Bone remodelling, Stress shielding, Neuromusculoskeletal modelling

## Abstract

**Introduction:**

The decision to retain or remove blade plate implants after proximal femoral osteotomy (PFO) in paediatric patients remains contentious. While retention provides ongoing support, it increases stress shielding, potentially hindering bone remodelling and causing long-term complications. Conversely, early removal may restore normal mechanical loading and promote bone recovery. This study compares the effects of blade plate retention versus removal on bone density changes and implant risk of yield (RoY) over 36 months in a paediatric femur.

**Materials and methods:**

A personalised neuromusculoskeletal modelling and finite element analysis framework was developed using computed tomography scans and gait data. Using a strain energy-based remodelling analysis, the framework assessed changes in bone density and RoY for two clinical participants, comparing intact femurs with those retaining the implant for three years or having it removed after one year.

**Results:**

In both participants, implant retention diminished proximal femur remodelling. In P1, the average proximal bone density with implant increased by 0.11 g/cm^3^ over 36 months, compared to 0.38 g/cm^3^ in the intact model. In P2, the intact model’s average proximal density increased by 0.27 g/cm^3^ versus 0.11 g/cm^3^ with the implant. Implant removal after 12 months reactivated remodelling, yielding final density changes of 0.14 g/cm^3^ (P1) and 0.21 g/cm^3^ (P2). The RoY decreased over time, stabilising at 71–75% for blade plates and 56–62% for screws.

**Discussion:**

These findings highlight the detrimental effects of prolonged retention due to stress shielding. Recovery in bone density after removal suggests that early removal may mitigate adverse effects and promote healthier bone adaptation, informing clinical decisions in paediatric PFO.

## Introduction

Paediatric bone remodelling is a dynamic and intricate process profoundly influenced by the skeleton’s rapid growth and developmental transformation characteristics. Surgical interventions such as proximal femoral osteotomy (PFO) can significantly affect this natural adaptation. PFO is the most common hip surgery in the paediatric population, utilised to correct deformities and enhance hip function [1, 2]. PFO is typically indicated in children with hip deformities such as developmental dysplasia, Perthes disease, or cerebral palsy, where realignment of the proximal femur is required to restore joint congruency and function [2]. However, given that PFO alters the mechanical environment of the femur, a lack of understanding of its impact on bone remodelling may limit the ability to predict long-term skeletal outcomes and optimise postoperative management.

One central aspect of PFO involves the use of an implant such as a blade plate, a device used for stabilising the bone fragments above and below the osteotomy site during the critical healing period post-surgery [1, 3–5]. While the blade plate provides important support during the initial bone union, its long-term presence raises significant questions regarding its impact on ongoing bone remodelling and overall bone health. Specifically, there is a lack of data on its stress-shielding effect on the paediatric bone.

Removing implants after fracture or osteotomy healing remains a topical issue in orthopaedics. The biomechanics of internal fixation are highly dynamic, with the continual development of newer and better fixation devices [6–8]. However, the criteria for implant removal have never been well-documented [9–12]. Reasons for retaining implants encompass continued mechanical support, though disadvantages include stress shielding, pain around the implant site, peri-implant fracture, and postoperative infection. Conversely, advantages of implant removal include restoration of physiological loading, reduced long-term implant-related complications, and simplified future surgeries [9, 13–16]. Whether implants are routinely removed also depends on healthcare system factors, with insurance coverage influencing decision-making. Alternatively, once bone union is achieved, removing the implant makes future surgeries less complicated, should they be required. Complications associated with implant removal include neurovascular injuries, bone fracture, and wound sepsis, with higher risks reported in procedures performed by less experienced surgeons [13, 16–18]. A survey of 655 orthopaedic surgeons revealed that 230 surgeons believed leaving an implant* in situ* poses a significant risk for subsequent implant fractures [9]. They also found that 37% of those surveyed said they would remove implants in patients under 40 years old, even in the absence of symptoms.

In children, it is generally believed that implants should be removed as soon as their functions have been achieved to avoid interfering with bone growth. However, there is no evidence in the current literature to support or refute the practice of routine implant removal in children [19–21]. In the context of PFO, the outcomes of removing or retaining the blade plate can alter the biomechanical environment, potentially impacting the natural process of bone remodelling and adaptation. This has been examined using mechanobiological frameworks such as Wolff’s law, which explains how bone adapts to mechanical stimuli. Though originally developed to describe physiological bone adaptation, Wolff’s law has also been applied in implant contexts to investigate how disrupted load transfer and stress shielding may suppress normal bone remodelling [22]. Wolff’s law, which describes the bone’s response to mechanical stimuli, remains vital for evaluating how surgical implants like blade plates influence paediatric bone remodelling processes. Building on this principle, finite element models that drive bone adaptation using local strain-energy density have become the standard approach for analysing implant-related remodelling in both adult and paediatric bones [23–29], yet translation of these simulations to clinical decision-making remains limited.

Clinical follow-up studies have documented peri-implant hip pain and radiographic changes consistent with stress shielding, as well as implant-related fractures, in children who retain blade plates beyond bony union; sometimes necessitating secondary procedures or prolonged rehabilitation [14, 30, 31]. Long-term consequences may include diminished femoral neck bone mass and altered proximal geometry, yet no consensus exists on the ideal timing of plate removal. Quantitative neuromusculoskeletal-finite element analysis (NMSK–FEA) simulations that predict subject-specific changes in bone density and implant stress therefore offer practical means to forecast risk, personalise follow-up, and support evidence-based decisions on early versus prolonged retention.

This study aimed to predict the biomechanical outcomes of blade-plate retention versus removal, using a personalised NMSK-FEA bone-remodelling workflow to simulate adaptation during a 36-month period following PFO. By simulating the biomechanical environment of the paediatric femur with and without the blade plate, we evaluated the average changes in bone density for 36 months post-surgery. Additionally, we evaluated the implant risk of yield (RoY), an indicator of the implant’s susceptibility to ductile fracture. We hypothesised that extended retention of the blade plate following bone union would result in a significantly reduced rate of bone density change but also facilitate a reduced risk of implant yield over time. The overarching goal of this study is to determine how blade-plate retention versus removal affects paediatric femoral bone remodelling and implant stress, thereby generating quantitative evidence to underpin future clinical guidelines.

## Methods

### Neuromusculoskeletal-finite element modelling

De-identified low-dose computed tomography (CT) scans from two participants (participant P1 = age 12 years, sex: female, height: 1.72 m; mass: 93.4 kg; participant P2 = age 14 years, sex: female, height: 1.61 m; mass: 44.7 kg) who took part in a medical device trial at our institution were used for this study. Both patients were part of a cohort that had post-operative imaging and gait data available. The CT scans, collected per the clinical management protocol of the treating surgeon, were acquired with a Somatom Force syngo VB10A scanner (SIEMENS, Munich, Germany). The scans had an average slice thickness of 1.5 mm, an inter-slice distance of 1.0 mm, and an in-plane pixel size of 0.97 mm. Ethical approval to access the medical imaging data was obtained from the institutional ethics committee (HREC/18/QRCH/161; GU Ref No: 2019/770), with written informed consent secured from the participant’s legal guardian for the participants to participate in the trial.

Gait analysis was performed for both participants at the Queensland Children’s Motion Analysis Service (QCMAS) in Brisbane, Australia. Using retroreflective markers placed on key anatomical sites [32], a 10-camera Vicon system (Oxford, UK) recorded motion at 200 Hz, capturing static and walking trials. Each walking trial included at least one complete stance phase of the affected leg, walking at a self-selected pace. Ground reaction forces were measured at 1000 Hz using AMTI force platforms (Watertown, MA, USA). Fourteen leg and hip muscles were monitored with wireless EMG sensors (Noraxon, Scottsdale, AZ, USA) at 1000 Hz, following SENIAM guidelines [33]. All procedures adhered to appropriate guidelines and regulations per the Declaration of Helsinki principles.

Our previous work has detailed the neuromusculoskeletal (NMSK) pipeline employed in this study [34]. The post-operative gait data were processed and formatted for the OpenSim modelling environment [35] using digital filtering techniques [36]. Muscle excitations were normalised to peak amplitudes for each muscle. The external biomechanics were modelled using the Rajagopal 2015 model template in OpenSim v3.3, scaled to each participant’s anthropometry [32, 35, 37]. Adjustments to tendon slack length, optimal fibre length, and maximum isometric force were made to account for participant-specific characteristics [38, 39]. Joint motions and loads were calculated using OpenSim inverse kinematics and dynamics toolboxes. Muscle moment arms, lengths, and lines of action were computed using the muscle analysis tool [40]. Internal biomechanics were modelled via the Calibrated EMG-Informed NeuroMusculoSkeletal Modelling Toolbox (CEINMS) toolbox [41], estimating muscle activations, forces, and joint contact forces in EMG-assisted neural mode. CEINMS computed lower limb muscle dynamics and joint contact forces, while optimising torque and EMG tracking [42]. Upon completing the NMSK modelling, muscle lines of action and three-dimensional hip, knee, and patellofemoral contact forces were transformed into the local femoral coordinate system using custom MATLAB scripts (Mathworks, MASS, USA). Muscle and joint contact forces were discretised into seven load steps and used as loading conditions for FEA.

Three-dimensional models of the intact, unaffected femur of the participant were reconstructed from CT scans using Mimics v25 (Materialise NV, Leuven, Belgium). The proximal and distal fragments of the affected implanted femurs were also constructed with Mimics v25, utilising segmentation, wrapping, and smoothing procedures. Concurrently, computer-aided design (CAD) models of the PediLoc^®^ LCBP implants (OrthoPediatrics, Warsaw, Indiana, USA) were developed from CT scans and stereolithography files provided by the manufacturer for surgical planning, employing a reverse engineering approach. Self-tapping cortical and locking cortical screws were modelled as basic cylindrical entities. The CAD model of the LCBP implant, self-tapping cortical screws, and locking cortical screws were then assembled with the proximal and distal femur models in 3-Matic using Boolean operations to create detailed post-surgical models.

The segmented bones were meshed with 2 mm triangular surface elements and converted to linear tetrahedral volume elements in Hypermesh v14.0 (Altair Engineering, Inc, Troy MI, United States). The LCBP implant and screws were meshed using 0.5 mm 8-node linear brick elements. A mesh sensitivity analysis confirmed the appropriateness of the element size and type for this study, as results were not influenced by bone mesh sizes below 2.5 mm [4, 34]. Participant-specific linear inhomogeneous material properties were assigned to the bone models based on the established correlation between Young’s modulus, apparent density, and CT grayscale values [43, 44]. Ten layers of density-modulus properties were associated with each femur model. A constant Poisson’s ratio of 0.3 was applied to the femur models, irrespective of variations in Young’s modulus [45].

**Fig. 1:**
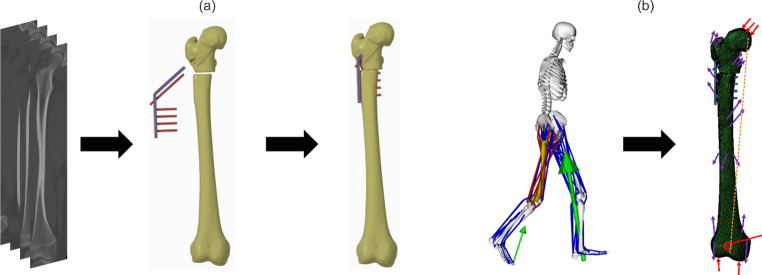
A post-operative NMSK-FE modelling pipeline was employed in this study. **a** Three-dimensional models of the femur and implant were created from post-operative CT images and assembled using a series of Boolean operations. **b** Joint contact and muscle forces were extracted from gait data collected approximately 9 months post-operatively and used as the loading conditions. The NMSK-FE model was completed by assigning participant-specific material properties, joint and muscle forces, and biomechanical boundary conditions

Subsequently, all models were exported to Abaqus Simulia v2022 (Dassault Systèmes, Simulia Corp, United States) for FEA. The implant and screws were modelled with material properties of medical-grade stainless steel, having a Young’s modulus of 190 GPa and a Poisson’s ratio of 0.33 [46, 47]. The interactions between screws and implants were defined using “tie” constraints, while tangential surface-to-surface contact interactions with penalty friction formulations were applied to other contact surfaces. Frictional coefficients were set at 0.3 for bone-implant interfaces and 0.46 for bone-bone interfaces, respectively [48, 49]. For both the intact and implanted femur, previously validated biomechanical boundary conditions were applied [34, 50], where an axial translational connector was established between the centre of the femoral head and the knee joint centre, where the hip contact force was subsequently applied. The knee joint centre was constrained in all translational degrees of freedom. Additionally, the most lateral aspect of the greater trochanter was restricted in the medial-lateral and anterior-posterior directions, as well as in rotation around the superior-inferior axis. A summary of the post-operative NMSK-FE pipeline developed for this study is presented in Fig. 1.

### Bone remodelling theory and analysis

In line with bone remodelling theories, apposition and resorption of bone tissues are driven by both external and internal remodelling processes, which significantly affect bone morphology and density [51]. The mechanical stimuli bone tissues detect are crucial in regulating this remodelling process. Various mechanical stimuli have been proposed over the years to explain long bone remodelling, with the strain energy density (SED) model being notably prominent [29, 52, 53]. The equations derived from using SED as the mechanical stimulus (*S*) were implemented in Abaqus CAE (Dassault Systèmes, Vélizy-Villacoublay, France) using the User-Defined Field (USDFLD) subroutine [54]. The SED per unit apparent density (*U*) and the local apparent density (*ρ*) are related by Eq. (1) [55]:

1 $${{S}}=\frac{{U}}{\rho}$$

The difference between the actual stimulus, *S*, and some reference stimulus, *S*_ref_, is assumed to be the signal responsible for bone resorption or formation. There exists a “lazy zone” or “dead zone” where no remodelling occurs [56]. This lazy zone is defined as:

2 $${{\left( {1 - x} \right){S_{ref}} \leqslant S \leqslant \left( {1+x} \right){S_{ref}}}}$$

where *x* denotes the tolerance of remodelling thresholds.

**Fig. 2:**
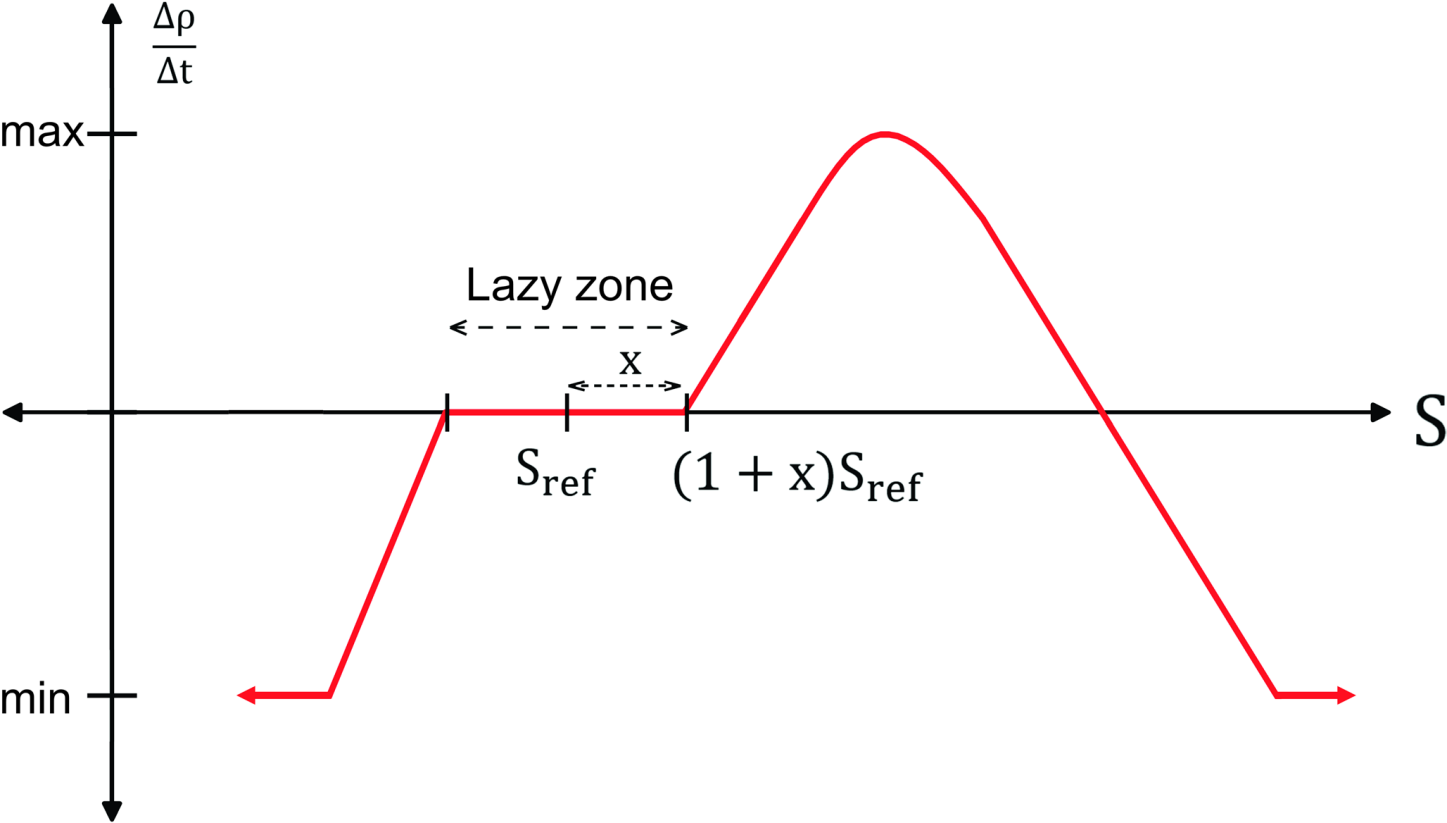
Graphical representation of the bone remodelling algorithm implemented in this study

Any remodelling stimulus outside the predefined lazy zone, defined by Eq. (2), will induce a change in bone density (∆*ρ*) governed by the remodelling rate constants, ${C}_{1}$ and ${C}_{2}$ Fig. 2. Implementing the remodelling rate constant and “lazy zone” into a single set of conditional equations to be evaluated by discrete time-based analysis, the general governing equation for bone remodelling becomes

3 $${{\frac{{\Delta \rho }}{{\Delta t}}}}=\left\{ {\begin{array}{*{20}{c}} {{C_1}\left[ {S - \left( {1+x} \right){S_{ref}}} \right] - {C_2}{{\left[ {S - \left( {1+x} \right){S_{ref}}} \right]}^2}}&\rm{for~~~S \geqslant \left( {1+x} \right){S_{ref}}} \\ {{C_1}\left[ {S - \left( {1 - x} \right){S_{ref}}} \right]}&\rm{for~~~S \leqslant \left( {1 - x} \right){S_{ref}}} \\ 0&\rm{otherwise} \end{array}}\right.$$

#### Numerical implementation

Equation (3) was consequently implemented iteratively in the Abaqus user subroutine USDFLD with the following constants: x = 10%, $\:{C}_{1}=60$ and $\:{C}_{2}\:=120$ (month x g/cm^3^), $\:{S}_{ref}=3.6\times\:{10}^{-5}\:$J/g [57, 58]. These constants were adopted from previous adult bone remodelling studies and are not specifically validated for paediatric bone tissue; however, they were applied consistently across all models to enable direct comparative analysis rather than to optimise paediatric-specific bone growth parameters. In its implementation, SED and the remodelling stimulus were calculated for each bone element at each simulation load step. These calculations, in turn, informed updates to the material properties of each element to reflect ongoing changes in bone density caused by mechanical loading. The simulation iteratively applied these updates for each of the successive seven load steps representing the full gait cycle as well as the necessary time steps representative of 36 months to capture the dynamic process of bone remodelling. During each bone remodelling iteration, for a given gait cycle step and time point, the algorithm assessed whether the changes in bone density and material properties across all elements were sufficiently small to indicate stability. Specifically, convergence was determined by evaluating the difference in strain energy density and material property updates between successive load steps. If these differences fell below a predefined threshold (± 0.05%), the solution was considered converged, meaning the bone density distribution had stabilised under the applied mechanical conditions. The algorithm continued to iterate until this condition was met, recalculating strain energy density and updating material properties.

**Fig. 3:**
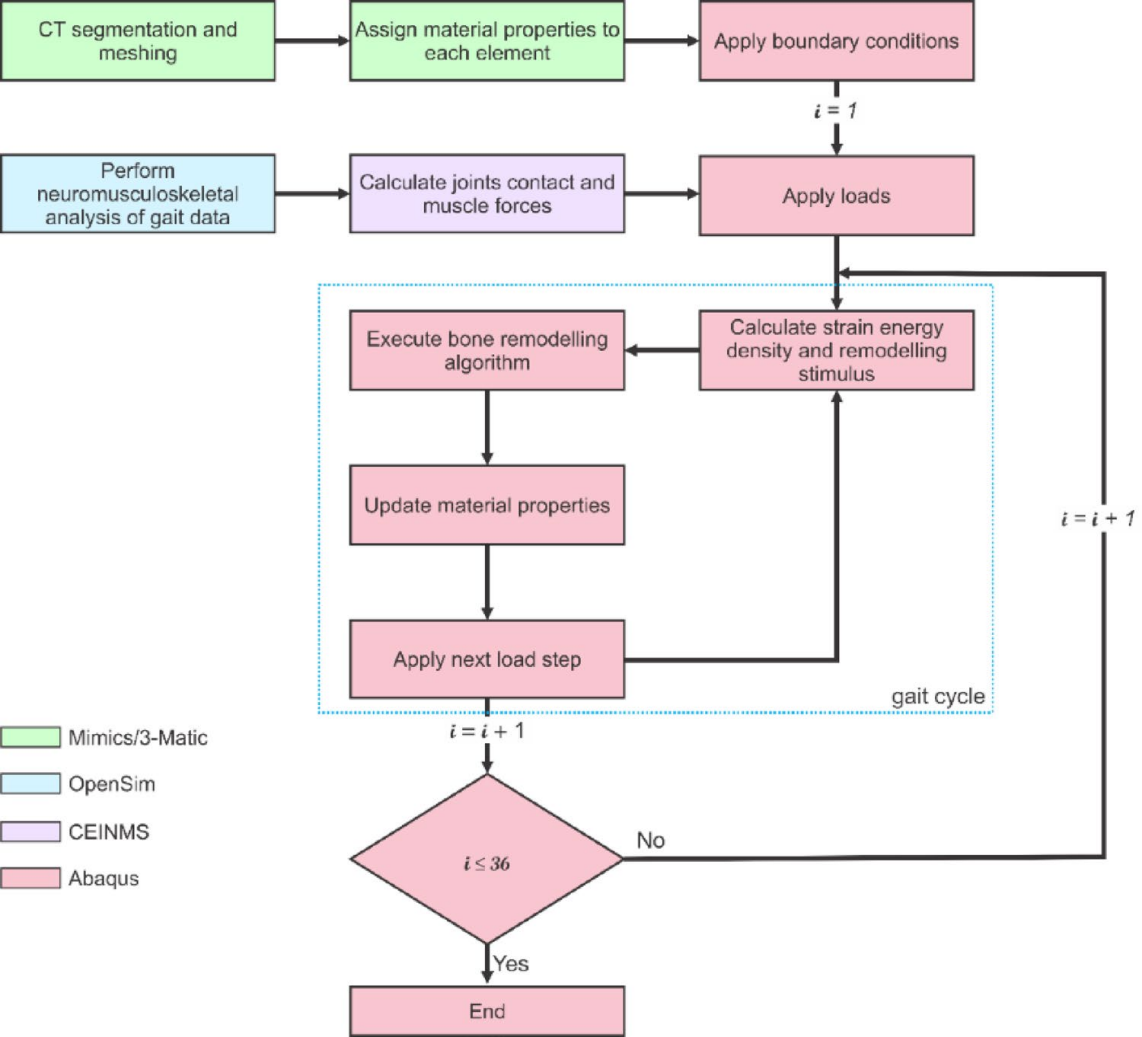
Neuromusculoskeletal-finite element models were created from gait and imaging data and used to implement the strain energy-based bone remodelling algorithm to investigate bone density changes over 36 months. 7 gait cycle load steps representative of one-month intervals are completed for each gait cycle

It should be noted that the time increment, $\:\varDelta\:t$, is an adjustable parameter and was selected to be a one-month interval representing the cumulative adjusted gait loading over that period. The constant $\:\rm{S}_{ref}$ matches the time period as previously presented by [58]. This iterative approach (simulated for over seven gait steps for each time interval and performed over 36 months) resulted in computational time of about 1 h for the intact models and 8 h for the implanted models on a 64 GB RAM Windows computer with an Intel core i9 processor on a 13-cpu run. Figure 3 summarises the implementation of the NMSK-FEA SED-based bone remodelling process.

### Simulated models

Three models were created and compared for each participant: intact, implant-retained, and implant-removed. The intact model was created from the contralateral leg, whereas the implant-retained and implant-removed models were created from the affected leg, as described in Sect. 2.1. The contralateral leg was selected for the intact model because paediatric hips are generally bilaterally symmetrical in morphology and bone density when no pathology is present, providing a reliable reference *in lieu* of a pre-operative scan. The applied loads were the same across the three models for each participant. The analysis for the intact models was simulated for 36 months to facilitate future comparisons for detecting stress shielding on lateral radiographs for future studies [59] and to reduce the computational costs involved with the simulation. The analysis for the implant-retained model was simulated for 12 months, after which the surface-to-surface contact between the proximal and distal fragments was changed to a “tie” contact to assume solid bone union [14, 60]. The analysis was then continued for another 24 months.

Similar to the implant-retained model, the analysis for the implant-removed model was initially run for 12 months. Following that, the implant and screws were deactivated/removed from the analysis. The proximal and distal cut surfaces were “tied,” assuming perfect fracture healing. The implant-removed model was then simulated for 24 months to complete the 36 months of postoperative walking activities applied to the intact and implant-retained models.

### Output measures

The average density changes in the proximal and distal bone fragments, along with the RoY for the blade plate and screws, were evaluated to assess the effect of implant presence on bone remodelling and implant failure susceptibility. Outcome measures were compared between intact and implanted models for each participant.

#### Average density change

The average change in bone density for the proximal and distal bone fragments was evaluated as a function of implanted time. Data output was produced in monthly intervals for the difference in density (from the analysis start to the end of the 7th load step for each iteration) for each element and then averaged over all elements (Eq. (4)).

4 $$\Delta {\rho _i}~=~\frac{1}{N}\mathop \sum \limits_{{k=1}}^{N} \left[ {\rho _{{i,7}}^{\rm{{end}}}\left( k \right) - ~{\rho ^{\rm{start}}}\left( k \right)} \right],~~i=1, \ldots ,~36,$$

where $\:{\rho\:}^{\rm{start}}\left(k\right)\:$and $\:{\rho\:}_{i,7}^{\rm{end}}\left(k\right)$ denote the density of the *k*th element in the distal or proximal bone fragment at the beginning of the analysis (i.e., the patient-specific mapped density distribution from the CT data) and end of the 7th load step in the *i*th iteration, respectively. *N* is the total number of elements in the designated region. This calculation was automated using an in-house Abaqus postprocessing Python script.

#### Implant risk of yield (RoY)

The RoY for the implant-retained model was determined by calculating the percentage ratio of the peak von Mises stress (PVMS) of the bone plate and screws to the yield strength of the implant material (Eq. (5)) (792 MPa) [47]:

5 $${\rm{Risk~of~yield~\left( {RoY} \right)=~\frac{{Peak~von~Mises~stress~\left( {PVMS} \right)}}{{Implant~material~yield~strength}}~ \times 100\%}}$$

The PVMS was utilised to calculate the RoY, capturing the highest stresses on the implant. PVMS has been empirically shown to align well with the actual yielding behaviour of many ductile materials [61], and as such this output measure represents the risk of initial implant failure in a conservative manner. An in-house script was developed to automate post-processing result extraction, specifically extracting PVMS while identifying and filtering out unrealistic, singularity-driven stress values. The script analysed the stress field and accepted maximum values within 10% of those at the integration points of neighbouring elements [62].

## Results

### Average change in bone density

**Fig. 4:**
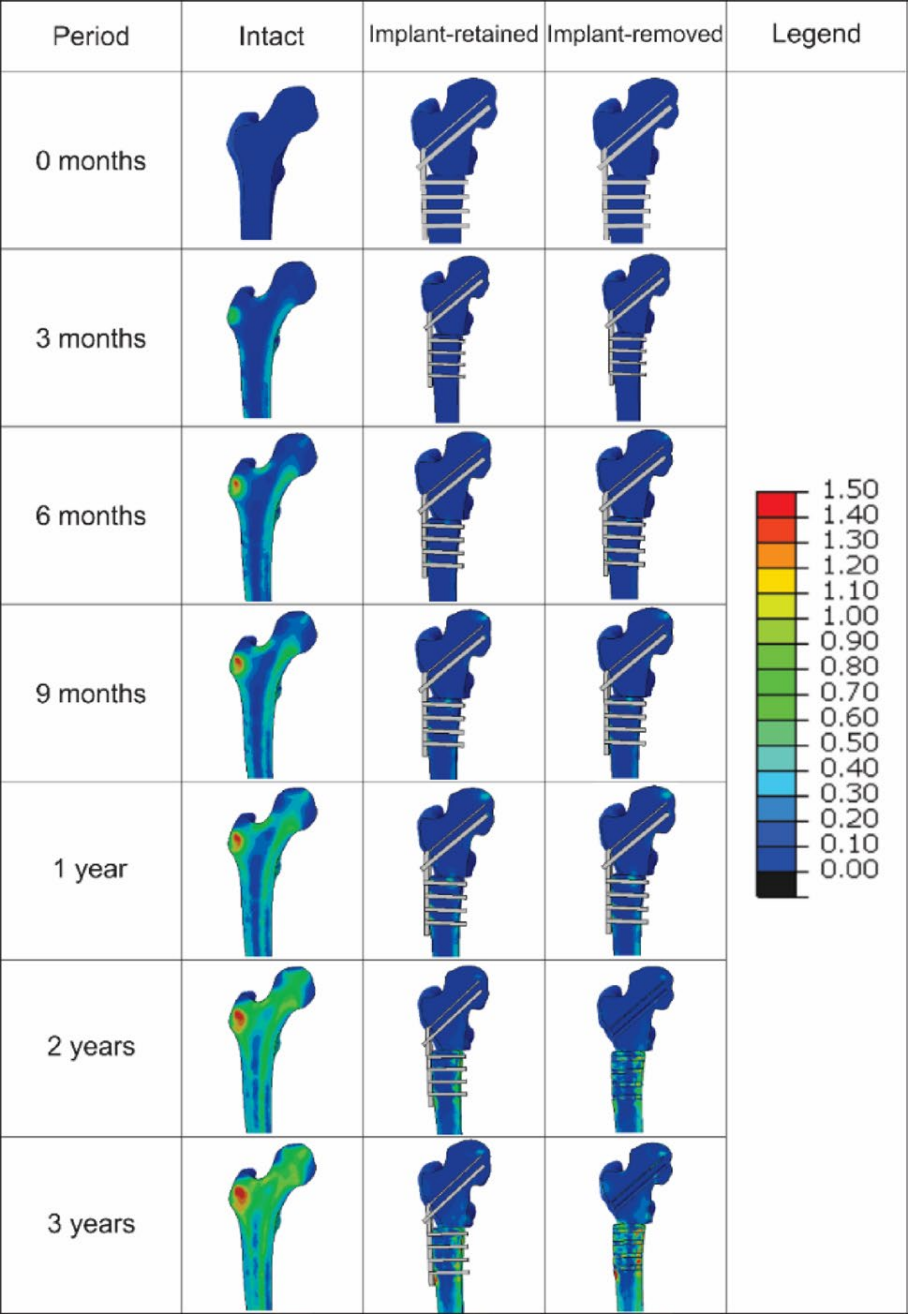
Contours depicting the element-based changes in bone density over three years for the intact, implant-retained, and implant-removed models for participant P1. Legend unit: Δ Density (g/cm³)

**Fig. 5:**
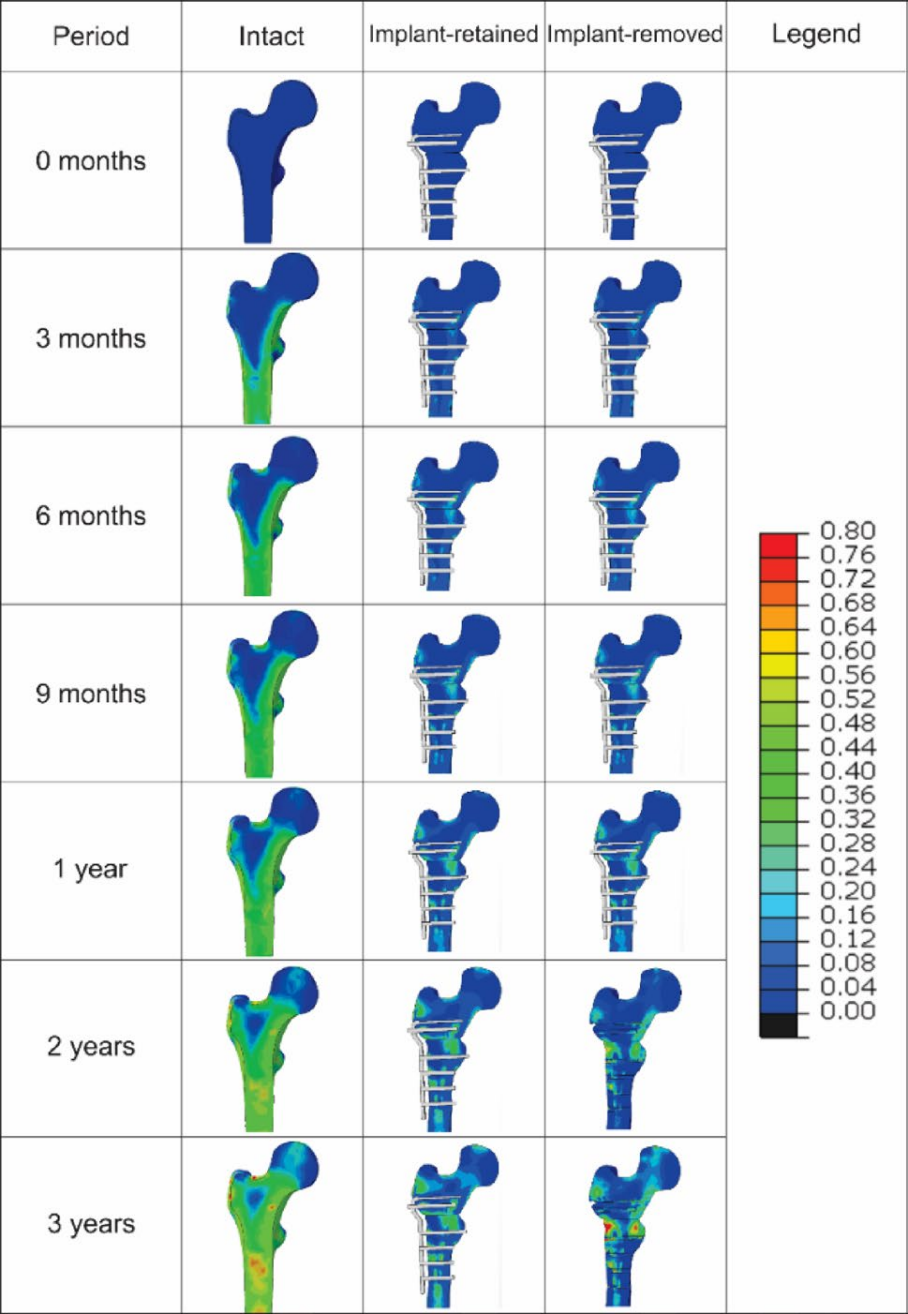
Contours depicting the element-based changes in bone density over three years for the intact, implant-retained, and implant-removed models for participant P2. Legend unit: Δ Density (g/cm³)

Figures 4 and 5 depict the simulated element-based bone density change distribution of the femur for the intact, implant-retained, and implant-removed models over the 3-year walking gait for participants P1 and P2, respectively. The contour plots show that the blade plate and screws altered the density pattern in both participants, and that mean density gains were 50–76% lower proximally and 50–56% lower distally when the plate was retained compared with the intact model. A comparison of P1 and P2 highlights that implant type and fixation depth also modulated the regional response. Plate removal at month 12 restored approximately one-quarter of the suppressed density by month 36, particularly in the distal region and along the osteotomy line, an effect more prominent in P2 (Figs. 4 and 5).

**Fig. 6:**
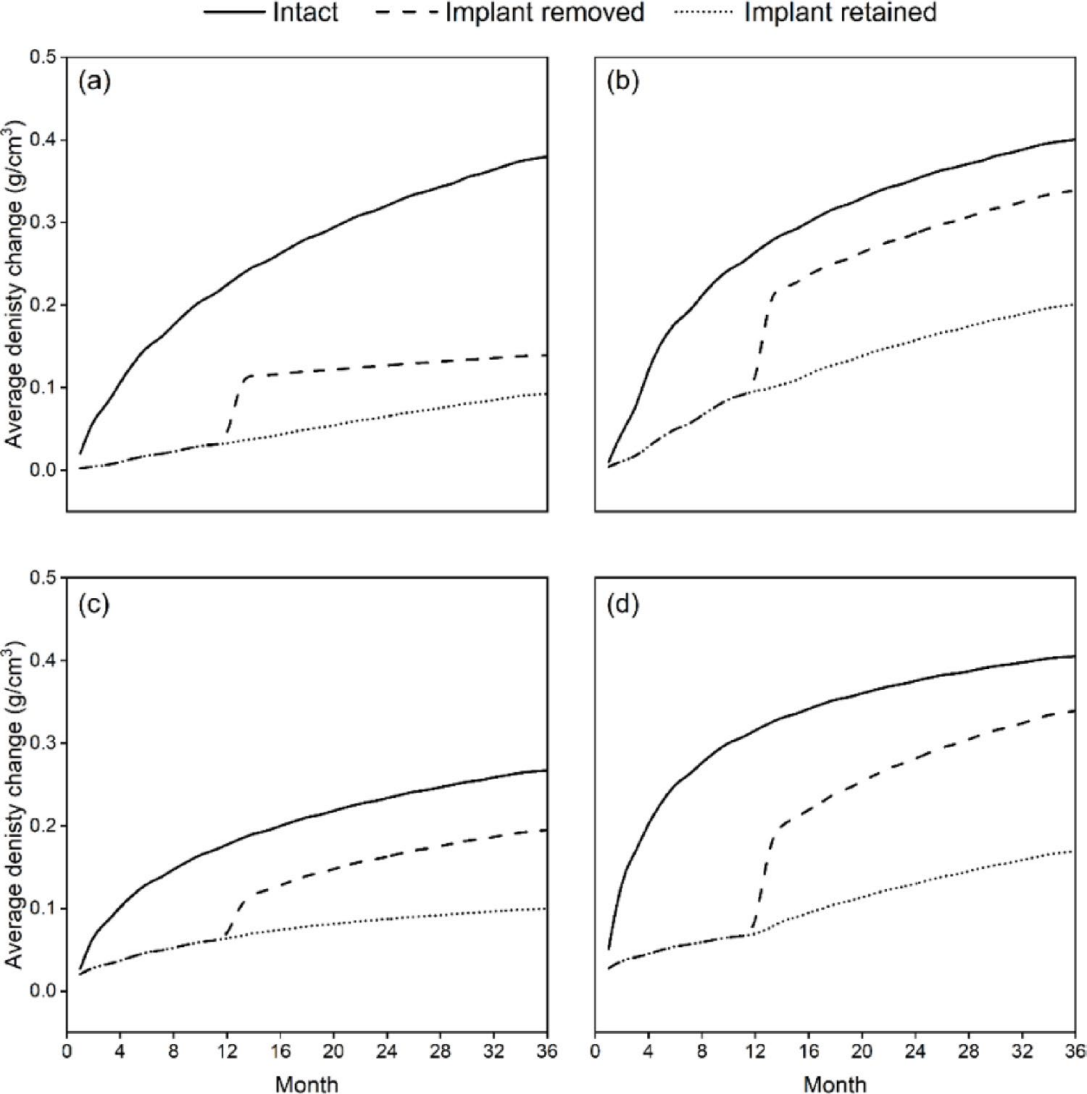
Average change in bone density (g/cm³) over 36 months during simulated walking gait. Panel (a) shows Participant P1, proximal femur; (b) Participant P1, distal femur; (c) Participant P2, proximal femur; (d) Participant P2, distal femur. Curves represent intact femur (solid line), implant removed (dashed line), and implant retained (dotted line).

For participant P1, in the intact bone model, the proximal section exhibited a monotonic increase in average density, beginning at 0.02 g/cm^3^ in the first month and progressively increasing to 0.38 g/cm^3^ at the end of the third year (Fig. 6a). For the implant-retained model, the proximal bone fragment displayed a significantly smaller change in density, starting at 0.002 g/cm^3^ and gradually rising to 0.09 g/cm^3^ by the end of the third year. However, upon removal of the implant after the twelfth month, an increase in density was observed, starting at 0.11 g/cm^3^ and continuing to rise to 0.14 g/cm^3^ by the end of the third year (Fig. 6a). The distal section of the intact natural bone also exhibited a consistent upward trend in density change, beginning at 0.01 g/cm^3^ in the first month and reaching 0.40 g/cm^3^ by the three-year mark (Fig. 6b). In the implant-retained model, the initial density change was 0.004 g/cm^3^, steadily increasing to 0.20 g/cm^3^ throughout the analysis. Following the removal of the blade plate implant after the twelfth month, there was a noticeable jump in density change from 0.09 g/cm^3^ to 0.21 g/cm^3^, after which the density continued to rise steadily, reaching 0.34 g/cm^3^ by the end of the third year as shown in Fig. 6. Density changes were non-uniform: elements directly under the lateral cortical portion of the plate and around screw heads showed ≤ 0.05 g/cm³ gain, whereas the medial cortex of the distal fragment reached ≥ 0.30 g/cm³ (Fig. 4).

The intact bone model’s proximal segment for participant P2 similarly demonstrated a steady and consistent rise in average density, beginning at 0.03 g/cm^3^ in the first month and increasing to 0.27 g/cm^3^ by the end of three years (Fig. 6c). In the implant-retained model, the proximal fragment exhibited a notably smaller density change, starting at 0.002 g/cm^3^ and gradually rising to 0.11 g/cm^3^ at the three-year mark. However, after the implant was removed following the twelfth month, the density increased more markedly, beginning at 0.12 g/cm^3^ and reaching 0.21 g/cm^3^ by the third year (Fig. 6c). The distal section of the intact bone similarly showed a steady upward trend, starting at 0.05 g/cm^3^ in the first month and culminating at 0.41 g/cm^3^ by the end of three years. In the implant-retained model, the initial density change was 0.03 g/cm³, steadily rising to 0.18 g/cm^3^ over the study period. After implant removal at the twelfth month, the distal segment’s density increased sharply from 0.07 g/cm^3^ to 0.18 g/cm^3^, continuing to grow steadily to 0.34 g/cm^3^ by the end of three years (Fig. 6d). A similar heterogeneous pattern appeared in P2. The region beneath the 90-degree plate and around the proximal screws demonstrated the smallest gains (< 0.05 g/cm³). Local resorption rings < 0.01 g/cm³ were also detected at individual screw–bone interfaces (Fig. 5).

### Blade plate and screws risk of yield (RoY)

**Fig. 7:**
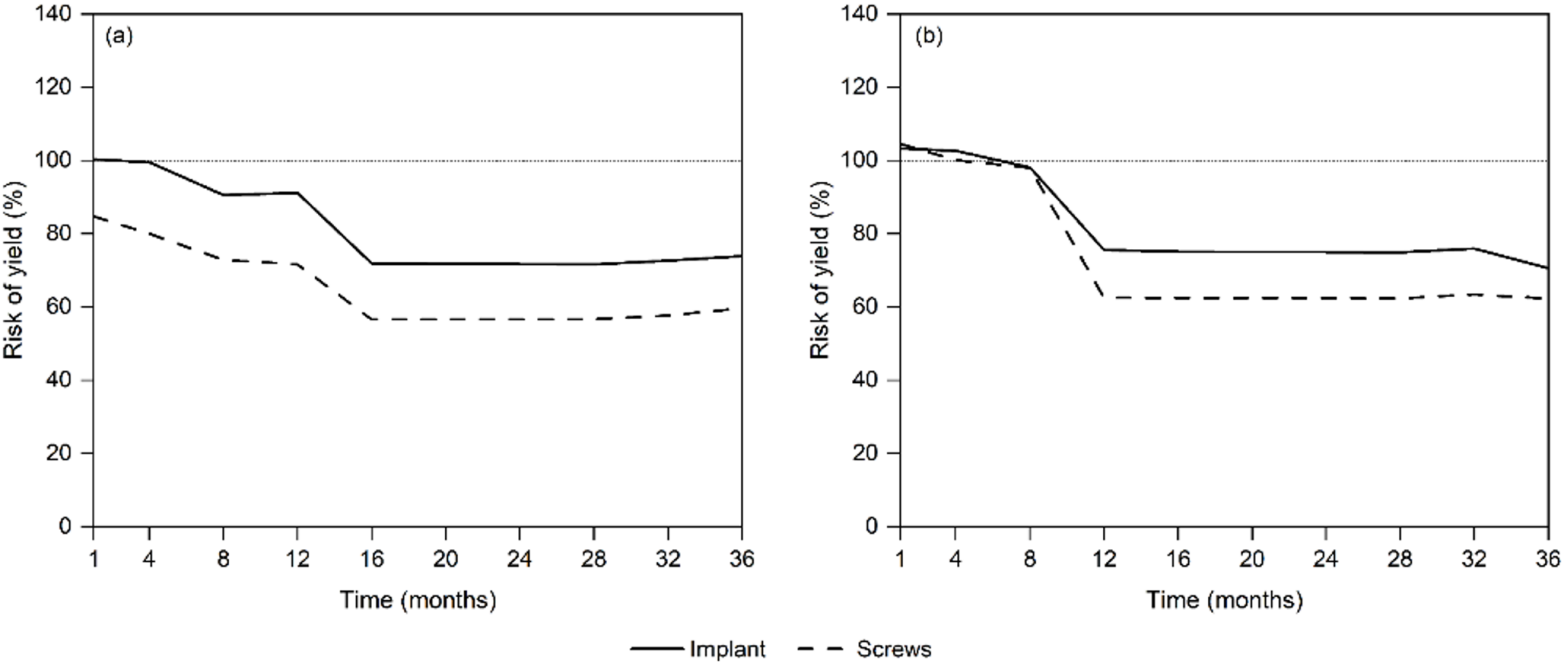
Risk of yield (RoY) for the blade plate (solid lines) and screws (dashed lines) in participants P1 (**a**) and P2 (**b**) across the 36-month simulation. The dotted line marks 100%, the threshold at which local yield is deemed to occur. For P1, RoY for both components declined steadily until month 16 and then stabilised. For P2, RoY fell until month 12, plateaued to month 32, and dropped further thereafter. During the first post-operative month, small cortical regions of the plate in both participants registered RoY values slightly above 100%, indicating possible incipient plastic strain; from month 2 onward all stresses remained within the elastic range (< 100%)

For participant P1, the RoYs for the blade plate and screw regions, respectively, were initially recorded one-month post-surgery at 100.25% and 84.72% respectively. The RoY of the implant showed a gradual decrease over the first 16 months, stabilising at approximately 72% from month 16 onward. Notably, there was a marked drop in blade plate and screw RoYs between months 12 and 16 (91.11–71.92% and 71.60–56.65%, respectively (Fig. 7).

For participant P2, at the outset, both the implant (103.27%) and screws (104.51%) displayed RoY values slightly above 100%, indicating a high initial stress. Between months 8 and 12, a notable reduction in the RoY for the implant occurred (98.10–75.59%), stabilising at approximately 75% from months 12 to 32, where further reduction in RoY occurred (Fig. 7). The screws showed a steeper decline, reaching approximately 63% by month 12, followed by a plateau in subsequent months with minimal variation, as shown in Fig. 7.

## Discussion

This study combines subject-specific gait loading, CT-derived heterogeneous bone material properties, and a strain-energy remodelling algorithm to deliver a novel, longitudinal NMSK–FEA comparison of blade-plate retention versus removal in paediatric PFO. The fully personalised pipeline captures the rapid, growth-related changes in paediatric bone morphology and load transfer that generic adult models overlook. By coupling bone-density adaptation with implant yield risk over a clinically relevant three-year horizon (while using the contralateral femur as an internal reference) the study links skeletal health and hardware durability within the same framework. This dual-output design yields actionable thresholds for both bone accrual and implant fatigue. At the same time, the workflow remains computationally tractable, making it feasible to scale to multiple patient cohorts in future analyses, to test alternative plate angulation, or to evaluate post-operative rehabilitation loads. Taken together, these strengths position the presented pipeline as a versatile in silico testbed that can accelerate evidence-based guidelines for the timing and strategy of implant removal in growing patients.

The findings of this study shows that subject-specific NMSK-FEA can quantify how blade-plate retention suppresses normal paediatric bone-density accrual and how removal partially restores it. In the tested cohorts the presence of the blade plate reduced proximal density gain by 50–76% and distal gain by 50–56% compared with the contralateral femur, while plate removal recovered ≈ 25% of the deficit within 24 months. Peak implant stresses dropped below the elastic-plastic threshold once bone union was achieved yet plateaued thereafter, signalling latent fatigue risk. These results establish a mechanistic link between stress shielding and blunted growth and demonstrate that a fully personalised workflow can detect clinically relevant trends even with a sample of two.

### Average change in bone density

The consistent increase in bone density observed in the intact femur model aligns with expected physiological responses, where mechanical loading during normal gait stimulates bone formation in the femur according to Wolff’s law [22]. The steady growth in bone density in the proximal and distal sections of the intact bone model reflects healthy bone adaptation in a paediatric skeleton, consistent with other studies [63, 64]. Therefore, NMSK-FEA models such as presented in this study can serve as a baseline against which the effects of the blade plate implant on bone remodelling can be evaluated.

The rising intact trace observed in this cohort is expected, as the baseline density field was mapped from CT scans obtained following surgery, when both participants were still pre-adolescent. Over the ensuing 36 months, physiological growth would naturally increase bone mass [65]. Within the strain energy–based framework, this ongoing apposition appears as mechanically driven adaptation and therefore a positive slope in the intact model. By contrast, a skeletally mature adult under load steady conditions would be expected to show a much flatter intact trajectory [65]. The significantly smaller average increase in bone density change observed in the proximal bone fragment of the implanted model when compared against the intact femur highlights the stress-shielding effect induced by the blade plate. The implant’s presence was found to alter the mechanical environment, reducing the load borne by the bone and consequently inhibiting the natural remodelling process [22, 66]. This markedly reduced increase in bone density over time is concerning, as it may predispose the bone to weaknesses, potentially increasing the risk of bone fracture both upon implant removal and later in life [9, 16]. The distal bone fragments in the implanted models exhibited a slower, less pronounced rate of density change, yet the change remained considerable when compared to the intact model. This outcome suggests that while the femur adjacent to the distal end of the blade plate is relatively less affected by stress shielding than the proximal segment in PFO, the implant’s influence remains sufficiently significant to impede normal bone adaptation.

Upon removal of the blade plate after one year, the immediate increase in bone density observed in both the proximal and distal sections indicates a reactive bone remodelling response. This increase, particularly in the bone fragment below the osteotomy, supports the well-accepted hypothesis that the removal of stress-shielding implants can partially restore the normal mechanical stimuli to the bone, thereby reactivating the bone remodelling process [67]. However, the post-removal density changes did not achieve the levels observed in the intact model, suggesting that the period of diminished mechanical loading during implant retention may impart lasting effects on bone health. The observed variation in the remodelling rate following implant removal for participant P1 may be attributed to the relatively larger implant size and type used. Specifically, while participant P2 utilises a 90-degree plate that does not extend into the femoral neck, P1 employs a 130-degree plate that traverses the femoral neck, potentially explaining the differences in remodelling dynamics. The change of implant placement in the femoral neck also resulted in distinct differences in density changes throughout this region (Figs. 4 and 5).

Adult finite-element studies of cementless hip stems report sustained proximal density losses of 20–35% five to ten years after implantation and only modest rebounds (0.05–0.10 g/cm³) following component revision, underscoring the long-term impact of stress shielding once growth plates are closed [59, 63]. By contrast, using remodelling constants adopted from adult literature [57, 58], the present paediatric models predicted net density gains of 0.14–0.21 g/cm³ within 24 months of plate removal. While the absolute magnitudes are influenced by the selected variables and differ from those in [59, 63] due to variations in remodelling formulations and parameter values, the proportional difference aligns with the faster adaptive capacity of immature bone described in other recent remodelling simulations [68]. This comparison confirms that, although the qualitative pattern of suppression during retention is shared, the magnitude of recovery is age-dependent and should be considered when timing implant removal.

### Implant risk of yield

The initially high RoY, especially evident at the one-month post-surgery mark, highlights the significant mechanical demands placed on the implant during the early stages of postoperative recovery. This observation aligns with the growing body of evidence advocating for careful post-surgical management, particularly during the critical phase before full weight-bearing is safely achievable [69, 70]. Although the RoY consistently decreased over the initial 12 months during gait loading, this reduction may not fully account for the mechanical challenges introduced by more demanding activities. High-impact or repetitive activities such as running or jumping could impose additional stresses on the implant, potentially accelerating wear or altering its long-term biomechanical performance.

It is important to note that the reported RoY remained relatively stable following implant retention for time periods greater than 12 months. Such a scenario would be expected to heighten the risk of implant failure under prolonged loading due to accumulated low-cycle fatigue [71], even if the overall yield risk appeared unchanged. Although RoY values for both participants stabilised below the static yield threshold, they remained within the 55–75% range that has been associated with fatigue crack initiation in 316 L stainless steel after approximately 10⁴ loading cycles [71]. These findings are particularly relevant in the context of a growing paediatric skeleton, where ongoing bone remodelling and changes in anatomy can further influence implant stability and effectiveness. Previous adaptive FE analyses that accumulated 10⁴–10⁶ gait cycles revealed that stress shielding and cyclic loading act synergistically: trabecular resorption increases implant bending, which in turn elevates peak stresses and shortens fatigue life [67, 68]. Retrieval data corroborate this interaction, with proximal bone loss and occasional stem fractures reported after prolonged cyclic service [59]. These findings reinforce the need for planned cycle-based damage module so that future simulations can quantify how cumulative loading influences both density suppression and implant fatigue. Thus, while the initial reduction in RoY for gait activities is promising, further investigations are required to assess the implant’s performance under patient-specific loading conditions to ensure its long-term durability and structural integrity.

### Considerations for implant retention and removal

The decision to retain or remove a blade plate should balance structural safety against the adverse effects of stress shielding. The simulation results were designed to align with diagnostic tools familiar to paediatric orthopaedic surgeons, such as standard radiographs, DXA scans, and routine hardware checks. Therefore, they can act as two quantitative triggers that can guide elective removal once healing is confirmed. A proximal femur density that remains more than 0.15 g/cm³ lower than the contralateral side or a month-on-month gain less than 0.01 g/cm³ after radiographic union suggests ongoing stress shielding [72, 73]. If the implant shows no structural damage under these conditions, further implant retention may offer little benefit and could increase cumulative fatigue risk, making removal appropriate if union and alignment are stable. Conversely, if the implant remains intact and bone density accrual remains robust, retaining the implant poses little risk. These dual thresholds condense complex modelling output into practical, image-based markers that can be checked at ordinary follow-up visits without extra radiation or invasive tests and thus form a rational bridge between the personalised NMSK–FEA workflow and everyday surgical decision-making.

The results support considering elective blade-plate removal soon after radiographic union, because prolonged retention suppressed proximal density accrual by up to 76% and maintained implant stresses within a fatigue-sensitive range. Conversely, removal before complete union could destabilise the osteotomy, precipitate loss of correction or refracture, and therefore should be avoided; confirmation of solid healing is an essential prerequisite. Furthermore, future simulation work could be pivotal in this decision-making process by informing rehabilitation tasks and exercises that optimally stimulate bone growth following implant removal. For example, when virtual rehabilitation programmes are developed, loading progressions could be considered acceptable if the simulated peak implant stress remains below 50% of the implant material's yield strength (RoY ≤ 50%) and proximal bone accrues at least 0.01 g/cm³ per month, a rate associated with physiological growth under strains of 800–2000 µε. Loads that suppress density gains below 0.005 g/cm³ could be flagged for modification before clinical translation. Such simulations could also help identify safe loading protocols that slow the progression of bone loss during the implanted stage, ensuring that the bone maintains sufficient density and integrity until removal is feasible. These efforts could enhance rehabilitation strategies and improve long-term bone health outcomes by leveraging computational models alongside clinical insights.

### Limitations and future directions

It is essential to acknowledge the limitations of this study, as they provide crucial context for the findings and help to inform future research directions. Firstly, the limited availability of biomechanical data on paediatric PFO restricts experimental validation in this study. Nevertheless, the bone remodelling algorithm used in this study has been considerably validated for the adult femur, lending credibility to the comparative findings [74, 75]. Moreover, confidence in the predictions is supported by a tiered validation chain encompassing external joint loads, muscle forces, boundary conditions, and patient-specific material properties [32, 41–43, 55, 62]. Future research should prioritise longitudinal clinical studies to validate these computational predictions and refine guidelines for optimal implant management in paediatric patients. Power analysis indicates that enrolling roughly 12–15 paediatric participants will provide 80% statistical power (α = 0.05) to detect a clinically meaningful ≥ 15% difference in proximal-density accrual between retention strategies, while still accommodating anatomical variability and remaining computationally feasible. Secondly, this study did not account for other factors that affect bone remodelling and healing after surgery, such as changes in gait, blood supply, diet, and lifestyle. These well-established determinants, along with various chemical, genetic, and biological processes, significantly influence bone healing [76]. Consequently, while the absolute magnitudes of the simulated gains may not have been directly validated, the relative differences among the intact, implant-retained, and implant-removed models, the primary focus of this study, remain valid because all models share the same systemic assumptions. Implant-stress and fatigue predictions are unaffected by this simplification. Future work will seek to incorporate endocrine growth models and perfusion-based stimuli to improve absolute density forecasts without compromising computational efficiency. These factors should be integrated to improve predictive models for the multiscale modelling of bone structures: at the macro, micro, and mesoscale. Thirdly, this study did not analyse the risk of implant failure due to fatigue loading. While the plateaued trend observed in the RoY indicates a lowered likelihood of ductile deformations or fractures, it also suggests the potential for continuous fatigue accumulation within the implant over the later time period [71]. A thorough investigation into implant fatigue necessitates a dedicated analysis that includes a detailed assessment of the local stress distributions within both the implant and the screws. Finally, key participant differences, such as BMI, implant design, size, positioning, and gait, influenced the analysis. While results were generally similar, this study could not determine their individual contributions, highlighting the complexity of biomechanical interactions. Implant size and positioning may be particularly important, as our recent studies have shown that subtle variations in placement and width-to-femoral neck ratio significantly affect biomechanical surgical outcomes [4, 77]. These findings underscore the need for further investigation to disentangle the effects of participant-specific factors and to refine implant design and placement strategies for improved clinical outcomes.

## Conclusion and clinical significance

This study contributes to evidence supporting the clinical recommendation for early implant removal following PFO in paediatric patients. Our findings reveal that blade plate retention significantly impedes natural bone remodelling due to stress shielding, particularly in the proximal fragment above the osteotomy site in the proximal femur. Although implant removal after the initial healing phase reactivates bone remodelling, the rate of bone density change does not fully recover to levels observed in intact bone, highlighting the lasting impact of prolonged stress shielding. Thus, careful postoperative monitoring and the consideration of implant removal as soon as clinically feasible are essential for promoting optimal bone health and functional recovery in paediatric patients undergoing PFO. In addition, findings from this study may help inform healthcare policy and insurance frameworks, particularly in regions where implant removal is not routinely covered, by providing evidence on the clinical relevance and potential benefits of routine hardware removal.

## Data Availability

Data is provided within the manuscript.
